# Heregulin, a new regulator of telomere length in human cells

**DOI:** 10.18632/oncotarget.4964

**Published:** 2015-07-22

**Authors:** Javier A. Menendez, Miguel A. Rubio, Judith Campisi, Ruth Lupu

**Affiliations:** ^1^ ProCURE (Program Against Cancer Therapeutic Resistance), Metabolism & Cancer Group, Catalan Institute of Oncology (ICO), Girona, Spain; ^2^ Girona Biomedical Research Institute (IDIBGI), Girona, Spain; ^3^ Laboratory of Hematology Service, Institut d'Investigació Biomèdica Sant Pau, Hospital de la Santa Creu i Sant Pau, Barcelona, Spain; ^4^ Lawrence Berkeley National Laboratory, Life Sciences Division, Berkeley, CA, USA; ^5^ Buck Institute for Research on Aging, Novato, CA, USA; ^6^ Mayo Clinic, Department of Laboratory Medicine and Pathology, Division of Experimental Pathology, Rochester, MN, USA; ^7^ Mayo Clinic Cancer Center, Rochester, MN, USA

**Keywords:** heregulin, telomere, TRF2, RAP1, cancer, aging

## Abstract

The growth factor heregulin (HRG) promotes breast cancer (BC) tumorigenesis and metastasis and differentially modulates BC cell responses to DNA-damaging agents *via* its dual extracellular and nuclear localization. Given the central role of telomere dysfunction to drive carcinogenesis and to alter the chemotherapeutic profile of transformed cells, we hypothesized that an unanticipated nuclear function of HRG might be to regulate telomere length. Engineered overexpression of the HRGβ2 isoform in non-aggressive, HRG-negative MCF-7 BC cells resulted in a significant shortening of telomeres (up to 1.3 kb) as measured by Southern blotting of telomere terminal restriction fragments. Conversely, antisense-mediated suppression of HRGβ2 in highly aggressive, HRG-overexpressing MDA-MB-231 and Hs578T cells increased telomere length up to 3.0 kb. HRGβ2 overexpression promoted a marked upregulation of telomere-binding protein 2 (TRF2) protein expression, whereas its knockdown profoundly decreased TRF2 expression. Double staining of endogenous HRGβ2 with telomere-specific peptide nucleic acid probe/fluorescence *in situ* hybridization (PNA/FISH) revealed the partial localization of HRG at the chromosome ends. Moreover, a predominantly nucleoplasmic staining pattern of endogenous HRGβ2 appeared to co-localize with TRF2 and, concomitantly with RAP1, a telomere regulator that specifically interacts with TRF2. Small interfering RNA-mediated knockdown of HRG decreased the expression of TRF2 and RAP1, decreased their presence at chromosome ends, and coincidentally resulted in the formation of longer telomeres. This study uncovers a new function for HRGβ2 in controlling telomere length, in part due to its ability to regulate and interact with the telomere-associated proteins TRF2 and RAP1.

## INTRODUCTION

The isoform β2 of the heregulin (HRG) family of growth factors (HRGβ2) is a tumor-promoting growth factor conventionally described as an indirect activator of HER2 (erbB2) oncogene-driven signaling *via* its ability to bind HER3 (erbB3) and HER4 (erbB4) [1–8]. Our previous work showed that expression of HRGβ2 cDNA in estrogen-dependent MCF-7 BC cells is sufficient to promote the loss of estrogen dependence and the acquisition of resistance to anti-estrogens [9], two phenotypic features closely related to the malignant progression of BC. Indeed, HRGβ2 promotes the *in vivo* progression from an estrogen-dependent, antiestrogen-sensitive and non-metastatic phenotype to an estrogen-independent, antiestrogen-resistant and metastatic phenotype [9, 10]. Stable suppression of HRGβ2 in HER2-negative metastatic BC cells efficiently abrogates their intrinsically aggressive behavior by inhibiting cell proliferation, preventing anchorage-independent growth and reducing their invasive potential *in vitro* [11]. Moreover, HRGβ2 blockade is accompanied by a marked reduction in tumor formation, tumor size, and an absence of metastasis *in vivo* [11], thus confirming the ability of HRGβ2 to drive carcinogenesis independently of HER2.

HRGβ2 differentially modulates BC cell sensitivity to DNA-damaging agents [12–14]. Forced expression of HRGβ2 promotes hypersensitization of BC cells to doxorubicin (DOX), an inducer of DNA double-strand breaks (DSB). Conversely, HRGβ2 overexpression confers resistance to the alkylating agent cisplatin (CDDP). Because overexpression and hyperactivation of HER2 determines also the sensitivity profile of cancer cells to DNA-damaging drugs [15, 16], it could be contended that HRGβ2-driven BC chemosensitivity merely reflects an ability of HRGβ2 to activate HER2. Our previous studies, however, showed that the tumorigenic properties of HRGβ2, which depend largely on its ability to activate the HER2-/-3/-4 network, could be dissociated from its regulatory effects on chemosensitivity to DNA-damaging agents. Accordingly, a non-tumorigenic structural mutant of HRGβ2 lacking N-terminal sequences and the cytoplasmic domain was sufficient to enhance BC cell sensitivity to DOX while abolishing resistance to CDDP [13].

An attractive molecular candidate to explain the paradoxical effects of HRGβ2 on carcinogenesis and chemosensitivity is the telomere, and more specifically, its end-capping function. On the one hand, telomere length stability is one of the key factors contributing to the proliferative capacity of many cancer cell types; consequently, depending on their length and functional state, telomeres serve to suppress or promote malignant transformation [17–19]. On the other hand, the inhibition of telomere maintenance acts to chemosensitize cancer cells to DSB inducers (e.g., doxorubicin), whereas long telomeres are good targets for drugs targeting the G-rich telomeric sequence (e.g., cisplatin). Accordingly, telomere dysfunction has been shown to be a central molecular determinant governing the chemosensitivity of cancer cells to agents that induces DSBs including DOX [20, 21], while massive telomere shortening and degradation is an early event of CDDP-induced apoptosis [22, 23].

HRGβ2 has been demonstrated to exhibit a dual cellular localization. It can be secreted into the intercellular space of the epithelium, where it performs its well-characterized paracrine or autocrine functions, and it can also translocate to the nucleus in cancer cells [13, 24, 25]. It remains unclear, however, which functions are exclusively dependent on the activation of HER receptors and which are exclusively attributed to nuclear HRGβ2. Here we hypothesized that the nuclear localization of HRGβ2 might relate to its ability to regulate telomere length and function in human cancer cells. We report for the first time that HRGβ2 is a previously unrecognized modulator of telomere length maintenance through its ability to regulate telomere-associated proteins, and plays an essential role in protecting telomere integrity at chromosome ends [26–35]. The discovery of HRGβ2 as a new regulator of telomere length suggests that the tumorigenic and metastatic functions of HRGβ2 might be explained in terms of its nuclear occupation as a novel interactor of the telosome/shelterin complex in human telomeres.

## RESULTS

### HRGβ2 overexpression promotes telomere shortening in breast cancer cells

**Figure 1:**
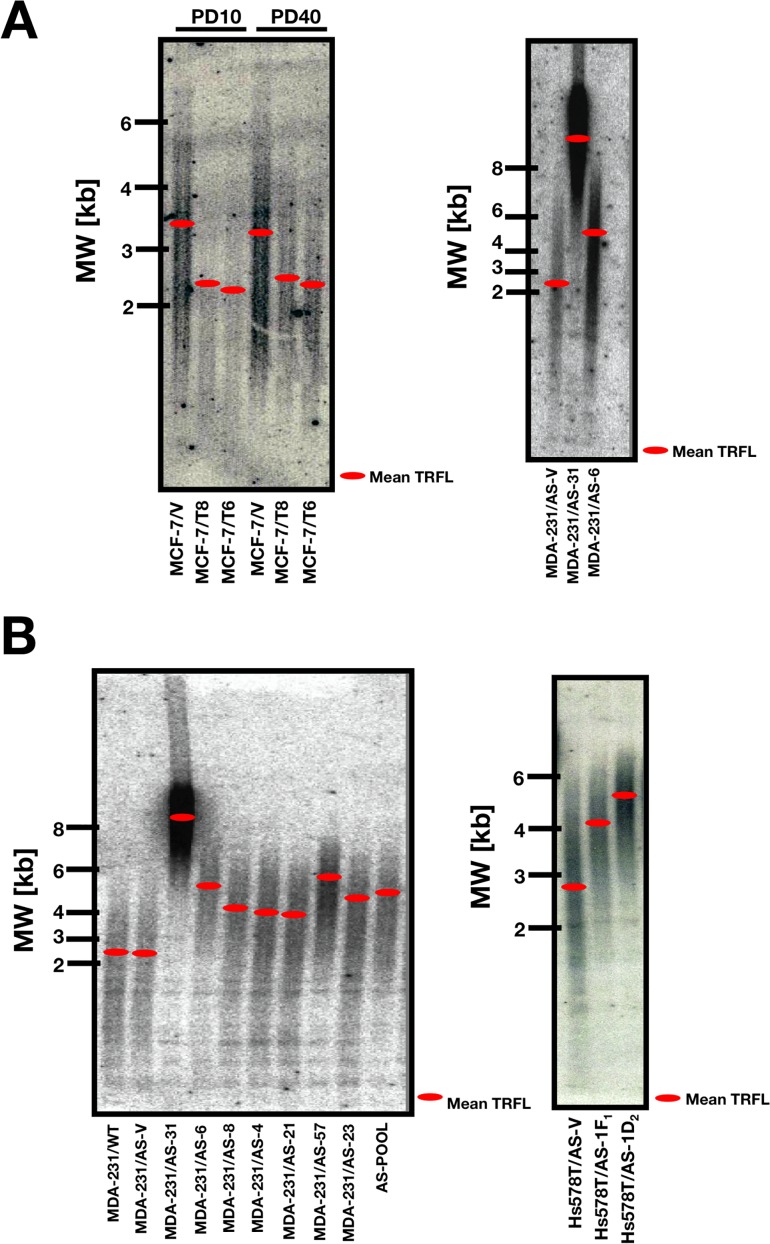
HRGβ2 regulates telomere length in human breast cancer cells **A.**, **B.** DNA from MCF-7 cells engineered to stably overexpress full-length human HRGβ_2_ cDNA (MCF-7/T clones) and equivalent empty-vector (MCF-7/V control cells), and DNA from HRGβ_2_-overexpressing MDA-MB-231 and Hs578T cells and equivalent antisense AS-HRGβ_2_ cells was isolated from collected cell pellets with inclusion of an RNase-A digestion step. Genomic DNA was digested with *Hin*fI/*Rsa*I, and genomic blotting for telomeric fragments was carried out according to standard protocols (see “Materials and Methods” section for details). In-gel hybridization was performed using an end-labeled (TTAGGG)_3_ probe following *in situ* denaturation of the DNA. Telomere gels were exposed to a Phosphorimager screen for 24 h and signals were quantified using ImageQuant software. *Red bars* indicate the median size of telomere length. The numbers on the left show the positions of a DNA-sizing ladder (in kb).

To study the effects of HRGβ2 forced expression on telomere length, we employed a BC progression model previously developed in our laboratory in which full length HRGβ2 cDNA is transfected into non-metastatic MCF-7 BC cells [9]. We used Southern blotting to evaluate the length of the terminal restriction fragments (TRF) in two representative HRGβ2-overexpressing MCF-7 clones (T6 and T8) and in control, empty vector cells. Although it is acknowledged that TRF length and telomere length are not the same measurements (telomere length is a measure of the terminal TTAGGG tract, whereas TRF reflects telomeres and adjacent sub-telomeric regions), TRF analysis can be used to determine the mean length of the terminal TTAGGG tract or the mean telomere length by measuring both the rate of decrease of mean TRF length and telomeric signal intensity as a function of population doubling [36, 37]. Using this measurement, we found that the average TRF length was significantly shortened from 3.4±0.2 kb in empty vector-transfected MCF-7 cells (MCF-7/V), to 2.4±0.1 and 2.2±0.1 kb in T6 and T8 clones, respectively (Figure 1A, *left panel*). No major changes in telomere length were observed over many ensuing population doublings

### Antisense inhibition of HRGβ2 promotes telomere lengthening in breast cancer cells

To validate the above findings, we utilized a cancer cell line that naturally overexpresses HRGβ2, and stably transfected an antisense (AS) HRGβ2 construct [11]. Two representative AS-HRGβ2 clones of the MDA-MB-231 human BC cell line (MDA-MB-231/AS-31 and MDA-MB-231/AS-6) exhibited marked differences in telomere length compared with vector control, as assessed by genomic blotting (Figure 1A, *right panel*). We previously demonstrated that, when the HRGβ2 protein expression was determined by Western blot analysis using an anti-HRG rabbit polyclonal antibody generated in our laboratory [9], the expression of HRGβ2 was significantly reduced by 25-30-fold in the AS-6 cells and it becomes almost undetectable in AS-31 cells as compared to empty vector-transfected MDA-MB-231 cells (AS-V) [11]. Average TRF length was significantly lengthened from 2.4±0.2 kb in MDA-MB-231/AS-V cells, to 5.5±0.2 kb in MDA-MB-231/AS-6 cells. Notably, telomeres in MDA-MB-231/AS-31 cells were considerably lengthened (8.6±0.5 kb), i.e., 6.2 kb longer than the mean telomere length in control cells. The mean TRF length in AS-6 and AS-31 clones did not vary over many ensuing population doublings.

To exclude the possibility that these observations were clone-specific, TRF length was evaluated in a wider panel of AS-HRGβ2 and MDA-MB-231 clones. The TRF length of control cells (MDA-MB-231/WT and MDA-MB-231/AS-V), seven single-clone AS-HRGβ2 transfectants (AS-31, AS-4, AS-6, AS-8, AS-21, AS-57, and AS-23), and an AS-HRGβ2-transfected pooled population (AS-POOL) are shown in Figure 1B (*left panel*). A significant increase in telomere length was observed in all single-clone AS-HRGβ2 derivatives (mean TRF length = 5.2 kb; range 3.9-8.6 kb) as well as in the AS-POOL population (TRF length = 5.0±0.2 kb). Similar results were obtained in AS-HRGβ2 clones from the highly metastatic and HRGβ2-overexpressing Hs578T human BC cell line (Figure 1B, *right panel*). Accordingly, AS-HRGβ2 Hs578T transfectants showed a significant increase in TRF length, from 2.8±0.1 kb in empty vector-transfected Hs578T (AS-V), to 4.2±0.2 and 5.3±0.2 kb in the 1F_1_ and 1D_2_ AS-HRGβ2 clones, respectively.

### HRGβ2 does not modulate telomerase activity in breast cancer cells

**Figure 2:**
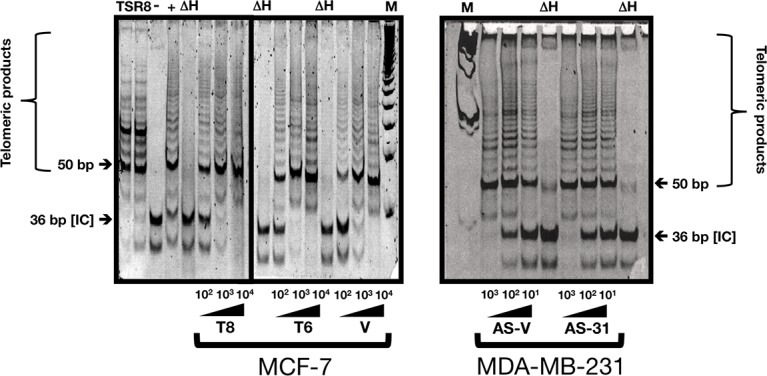
HRGβ2 does not modify telomerase activity in breast cancer cells Telomerase activity was assessed by the Telomere Repeat Amplification Protocol (TRAP) using a commercial kit. One to four thousand cell equivalents were used for each reaction with a representative experiment shown (*n = 3*). TSR8: Synthetic telomerase product. IC: Denotes the 36-bp internal control that serves to normalize sample-to-sample variation. M: Molecular weight markers. ΔH: Heat-inactivated control.

Because telomere length is controlled by a homeostatic mechanism that involves telomerase and several telomeric proteins, it was important to determine whether HRGβ2-related changes in telomere length reflected changes in telomerase activity and/or telomere-binding protein expression. Using a sensitive semi-quantitative PCR-based assay (TRAP) to measure telomerase activity, we failed to detect significant changes in telomerase activity in HRGβ2-overexpressing MCF-7/T6 and MCF-7/T8 clones when compared with MCF-7/V control cells as assessed by the intensity of telomerase products detected in the TRAP assay (Figure 2, *left panel*). Similarly, we found no significant differences in telomerase activity of AS-HRGβ2 MDA-MB-231/AS-31 and MDA-MB-231/AS-6 clones when compared with MDA-MB-231/AS-V cells (Figure 2, *right panel*). Moreover, telomerase activity levels in Hs578T/AS-1F_1_ and Hs578T/AS-1D_2_ clones did not differ when compared with Hs578T/AS-V cells (data not shown).

### HRGβ2 regulates TRF2 expression in breast cancer cells

**Figure 3:**
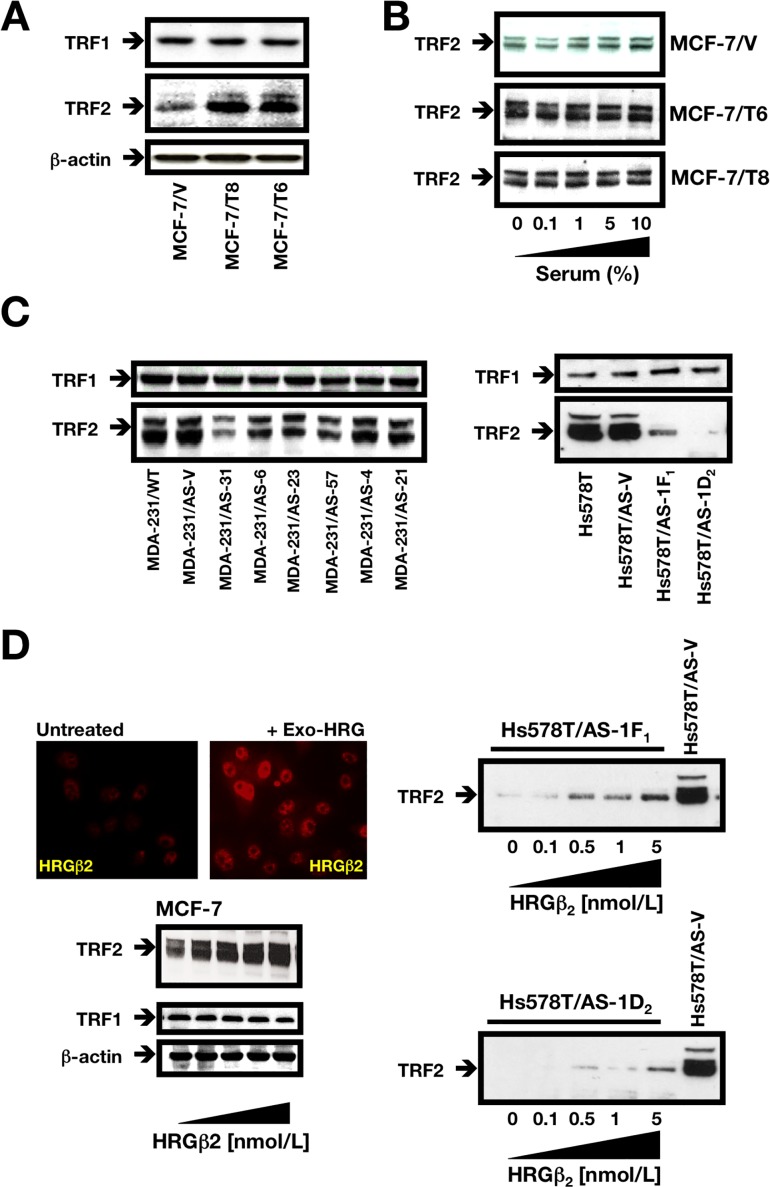
HRGβ2 regulates TRF2 expression in breast cancer cells **A.**, **B.** Cells were washed with cold PBS and solubilized in lysis buffer containing phosphatase and protease inhibitors. Fifty μg of protein per sample was resolved by SDS-PAGE and subjected to Western blotting for TRF1 and TRF2. One representative immunoblot is shown (*n = 3*) [A: Asynchronous populations of MCF-7/V, MCF-7/T6 and MCF-7/T8 cells growing in regular medium; B: Overnight serum-starved (60-70% confluence) MCF-7/V, MCF-7/T6 and MCF-7/T8 cells were cultured in the presence of increasing concentrations of FBS (0%, 0.1%, 1%, 5% and 10%) for 48 h]. **C.** Overnight serum-starved (75% confluence) MDA-MB-231 (*left*) and Hs578T (*right*) cell lines and equivalent antisense AS-HRGβ_2_ derivatives were subjected to Western blotting for TRF1 and TRF2. One representative immunoblot is shown (*n = 3*). **D.** Overnight serum-starved (75% confluence) MCF-7 (*left*) Hs578T/AS-1F1 and Hs578T/AS-1D2 cells (*right*) cells were treated with graded concentrations of recombinant HRG for 48 h in low-serum-medium (0.1% FBS). Cells were then washed with cold PBS and solubilized in lysis buffer containing phosphatase and protease inhibitors. Fifty μg of protein per sample was resolved by SDS-PAGE and subjected to Western blotting for TRF2. One representative immunoblot is shown (*n = 3*). A representative immunofluorescent image of MCF-7 cells stained for HRGβ_2_ before and after exogenous treatment with recombinant HRG (red) is shown.

Although several proteins occupy the mammalian telomere and serve to regulate its structure, two closely related proteins TRF1 and TRF2 (telomere repeat binding factor 1 and 2), appear to be exclusively localized to the mammalian telomere [26, 28]. TRF1 and TRF2 each bind double-stranded telomeric DNA as homodimers; TRF1 homodimers are postulated to monitor telomere length, while TRF2 homodimers serve to stabilize telomeric loop (t-loop) formation and protect telomere integrity. Immunoblotting of whole cell lysates from BC cells overexpressing HRGβ2 (MCF-7/T clones) using specific antibodies against TRF1 and TRF2 revealed that whereas TRF1 expression was unchanged relative to MCF-7/V control cells, TRF2 expression was markedly increased (Figure 3A). Indeed, expression levels of the 65/69 kb TRF2 doublet were significantly greater in HRGβ2 overexpressing cells regardless of whether the cells were actively cycling or growth arrested by serum starvation (Figure 3B).

When we compared TRF2 expression in AS-HRGβ2 clones with that found in naturally HRGβ2-overexpressing MDA-MB-231 and Hs578T cell lines, we observed a negative correlation between TRF2 expression and telomere length in AS-HRGβ2 MDA-MB-231 transfectants (Figure 3C, *left panel* and Figure 1B). Thus, MDA-MB-231/AS-HRGβ2 clones with longer telomeres had lower levels of endogenous TRF2 (e.g., AS-31, AS-6, and AS-57 clones in Figure 3C), whereas MDA-MB-231/AS-HRGβ2 clones with shorter telomeres had higher levels of endogenous TRF2 (e.g., AS-4 and AS-21 clones in Figure 3C). Moreover, TRF2 protein expression was dramatically reduced upon AS-driven blockade of HRGβ2 expression in Hs578T cells (Figure 3C, *right panel*). Indeed, we found TRF2 levels to be at least 90% lower in Hs578T/AS-1F_1_ and Hs578T/AS-1D_2_ clones relative to Hs578T/AS-V control cells (Figure 3C, *right panel*). Conversely, AS-driven down-regulation of HRGβ2 expression in MDA-MB-231 and Hs578T cells did not significantly affect TRF1 expression (Figure 3C).

As a complementary approach, we examined TRF2 expression after exposure of cells to recombinant HRG. Exogenous addition of HRG to HRGβ2-negative MCF-7 cells induced a specific and dose-dependent upregulation of TRF2 without affecting TRF1 levels (Figure 3D, *left panel*). Furthermore, exogenous HRG supplementation could partially recover the extremely low levels of TRF2 induced by AS-driven blockade of endogenous HRGβ2 in the AS-HRGβ2 Hs578T derivatives 1F_1_ and 1D_2_, (Figure 3D, *right panel*).

We next determined whether HRGβ2-related changes in TRF2 protein expression correlated with changes in TRF2 transcript levels. Thus, we extracted total RNA from MCF-7 cells overexpressing HRGβ2 and also from AS-HRGβ2 MDA-MB-231 and Hs578T transfectants, and measured TRF2 mRNA by RT-PCR. Results showed that, contrary to the marked differences observed in TRF2 protein levels, TRF2 mRNA levels were relatively constant between the different cell types and no correlation was found with TRF2 protein levels (data not shown).

### HRGβ2 localizes at telomeres with the telomere-associated proteins TRF2 and RAP1

**Figure 4:**
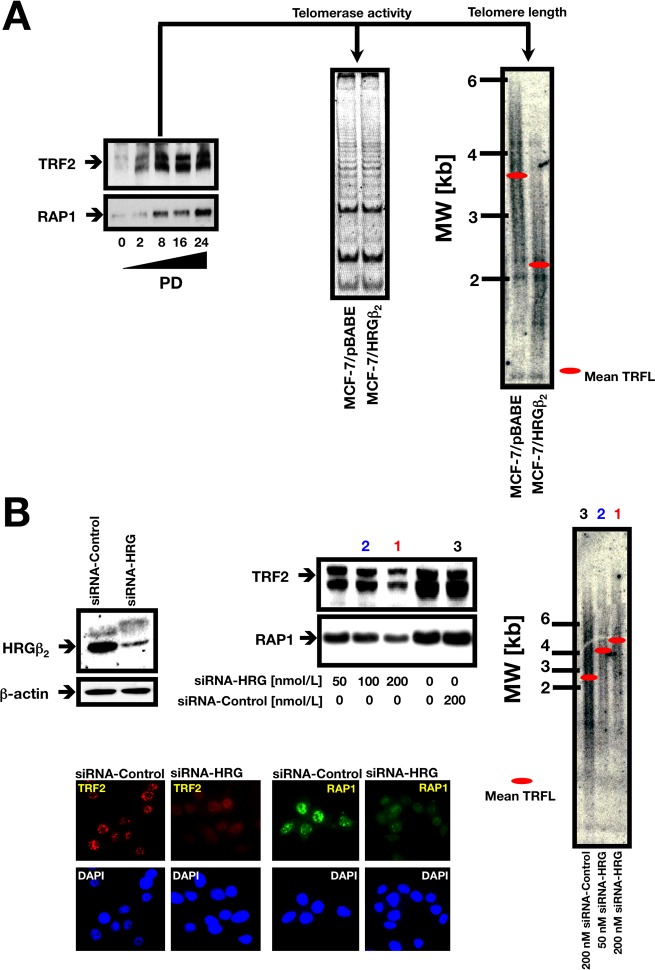
**A.** Retrovirally-induced HRGβ2 regulates TRF2 and RAP1 expression and telomere length in MCF-7 breast cancer cells. *Left.* Representative immunoblot (*n = 3*) showing upregulation of TRF2 and RAP1 in MCF-7 cells retrovirally engineered to overexpress HRGβ_2_. PD 0 represents the first sub-passage after selection for retroviral infection. At the indicated PDs, cells were washed with cold PBS and solubilized in lysis buffer containing phosphatase and protease inhibitors. Fifty μg of protein per sample was resolved by SDS-PAGE and subjected to Western blotting for TRF2 and hRap1 as described above (see “Materials and Methods” for details). *Middle panel.* MCF-7/HRGβ_2_ cells do not exhibit significant changes in telomerase activity as assessed by the TRAP assay. Three thousand cell equivalents were used for each reaction and a representative experiment is shown (*n = 3*). *Right panel.* Telomere length changes in MCF-7 cells upon retrovirally-induced HRGβ_2_ overexpression. The panel shows a representative genomic blotting analysis of telomeric restriction fragments in *Hin*fI/*Rsa*I-digested genomic DNA from retrovirally-infected MCF-7 cells probed with a TTAGGG repeat fragment. **B.** HRG knockdown reduces the presence of TRF2 and RAP1 on telomeres and promotes telomere lengthening. *Top left.* Depletion of HRG with siRNA significantly affects TRF2 and RAP1 protein levels. A representative Western blot of MDA-MB-231 cell lysates 72 h after transfection of siRNA to HRG or siRNA control is shown (*n = 3*). *Top right.* TRF length analysis in HRG knockdown MDA-MB-231 cells. The panel shows a representative genomic blotting analysis of telomeric restriction fragments in *Hin*fI/*Rsa*I-digested genomic DNA from MDA-MB-231 cells transiently transfected with control siRNA or graded concentrations of HRG siRNA (PD 4) probed with a TTAGGG repeat fragment (*n = 3*). *Bottom.* Reduced TRF2 and RAP1 telomeric signals after HRG siRNA treatment. Western blot analysis showing the significant depletion of HRGβ_2_ protein with a specific siRNA. The panel shows representative immunofluorescence images of MDA-MB-231 cells 48 h after introduction of HRG siRNA: staining with anti-TRF2 (*red*) and anti-RAP1 (*green*) antibodies is shown (*n = 3*). DAPI was used to visualize nuclear DNA (*blue*).

Finally, to further evaluate a causal role of HRGβ2 in telomere homeostasis, we undertook a multi-step approach. First, to better determine the consequences of increased HRGβ2 expression for both telomere length dynamics and telomere-binding protein expression, we introduced additional copies of HRGβ2 into HRGβ2-negative MCF-7 BC cells to achieve levels more consistent with those found in tumor-derived human cell lines. Thus, HRGβ2 expression was driven by a retroviral LTR instead of the CMV promoter employed in the generation of the T6 and T8 clones, to assure a sustained high level of expression. Although MCF-7 cells, akin to other human tumor cell lines, can exhibit a high degree of variation in telomere length and dynamics, the analysis of large pools of retrovirally-infected cells is known to reduce outlier effects, thus allowing a more consistent assessment of the impact of ectopically overexpressed proteins on telomere length. By examining a pool of HRGβ2-infected cells subjected to puromycin selection, we found a significant upregulation of TRF2 and RAP1, a protein involved in the regulation of telomere structure, as early as PD 2 (Figure 4A, *left panel*), and these levels were sustained at late PD cultures. We failed to detect significant changes in telomerase activity between MCF-7/HRGβ2 cells and MCF-7/pBABE matched control cells by TRAP assay (Figure 4A, *middle panel*); however, the average TRF length was significantly shortened from 3.6±0.1 kb in empty vector-infected MCF-7/pBABE cells, to 2.2±0.1 kb in MCF-7/HRGβ2 cells, as measured by Southern blotting (Figure 4A, *right panel*).

Having established that HRGβ2 can regulate TRF2 and RAP1 protein levels while concomitantly affecting telomere length maintenance, we next questioned whether HRGβ2 might modulate telomere homeostasis by forming a proteinaceous link with TRF2 and RAP1. To test this, we used siRNA transfection to deplete HRGβ2. Transfection of MDA-MB-231 cells with increasing concentrations of HRG-specific siRNA during 72 h resulted in a decrease of up to 70% of the baseline levels of HRGβ2 found in control (scramble siRNA) cells (Figure 4B). Of note, depletion of endogenous HRGβ2 diminished the abundance of TRF2 and RAP1 in a dose-dependent manner as assessed by immunoblotting. Further, immunocytochemistry confirmed a strong reduction in the telomeric signals of TRF2 and RAP1 upon HRGβ2 depletion since treatment with HRG-siRNA promoted an evident loss of the characteristic punctate pattern of TRF2 and RAP1 staining in 70% of the cells, and greater than 80% of the nuclei showed diminished TRF2 and RAP1 telomeric signals (Figure 4B).

Because a decrease in the accumulation of TRF2 and RAP1 upon HRGβ2 depletion might suggest that the stability and telomere-length regulatory functions of TRF2 and RAP1 are linked to the presence of HRGβ2 at chromosome ends, we next assessed the possible changes in telomere length in HRGβ2 knockdown cells. We observed that siRNA knockdown of HRGβ2 was coincidental with a significant increase in telomere length, from 2.6±0.2 kb in MDA-MB-231 cells transfected with scramble control siRNA, to 5.1±0.2 kb in MDA-MB-231 cells transfected with 200 nmol/L of siRNA duplexes against HRG (Figure 4B). No significant changes in telomerase activity could be detected upon silencing of HRGβ2 expression in MDA-MB-231 cells (data not shown).

**Figure 5:**
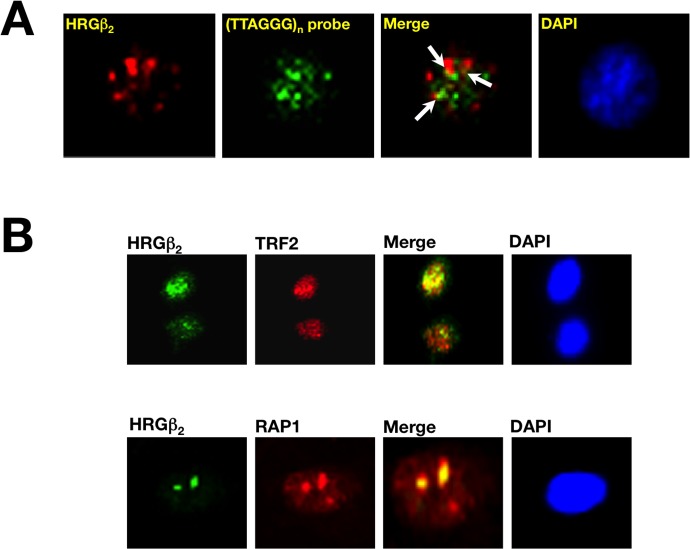
Telomeric localization of HRGβ2 in human cancer cells **A.** Subnuclear localization of HRGβ_2_ and its relationship with telomeric loci. A431 cells naturally overexpressing HRGβ_2_ were permeabilized twice and sequentially stained with an antibody against HRGβ_2_ (*red*) and the fluorescein-conjugated telomere-specific peptide nucleic acid (PNA) probe (*green*). DAPI was used to visualize nuclear DNA (*blue*). Merged images reveal partial co-localization of endogenous HRGβ_2_ and telomeres (*yellow*). **B.** Representative immunofluorescent images of A431 cells co-stained for endogenous HRGβ_2_ (*green*) using anti-TRF2 or anti-RAP1 (*red*) antibodies is shown (*n = 3*). Merged images, including DAPI staining of DNA, reveal a prominent co-localization (*yellow*) of endogenous HRGβ_2_ with TRF2 and RAP1.

Lastly, we evaluated the sub-cellular distribution of endogenous HRGβ2 and its spatial relationship with telomeres and telomere-binding proteins TRF2 and RAP1. To study this issue, and to demonstrate further that the functional association between HRGβ2 expression and telomere homeostasis was not a specific feature of the BC models used for these experiments, we employed the A431 cell line, an independent *in vitro* human epidermoid metastatic cancer model naturally overexpressing HRGβ2. Indirect immunofluorescence studies with an anti-HRGβ2 antibody confirmed a predominant nuclear accumulation of endogenous HRGβ2 in A431 cells. Moreover, FISH analysis with a peptide nucleic acid (PNA)-telomere probe on cell metaphases previously stained for HRGβ2, showed that nuclear HRGβ2 was able to partially co-localize with PNA-stained duplexes (Figure 5). Also, immunostaining with anti-TRF2 and anti-RAP1 antibodies demonstrated that the speckled distribution pattern of endogenous HRGβ2 was found to significantly co-localize with native TRF2 and RAP1 (Figure 5). Equivalent results were observed in MDA-MB-231, Hs578T and MCF-7/HRG cells (data not shown).

## DISCUSSION

We here present the first evidence that HRGβ2, a member of the HRG family of growth factors, is a novel regulator of telomere length in human cells. Our findings clearly show that HRGβ2 might negatively regulate telomere length by determining the ability of the telomere-binding proteins TRF2 and RAP1 to stably localize at chromosome ends.

Because telomere length in a cell population is determined by several factors including the activity of telomerase, the rate of telomere shortening, and the levels of telomere length controlling factors, we evaluated whether HRGβ2-induced changes in each of these parameters might explain the ability of HRGβ2 to reset telomere length to a new equilibrium. Using the classical PCR-based TRAP assay, we failed to detect any significant change in telomerase activity in response to changes in HRGβ2 expression. We therefore speculated that HRGβ2-induced changes in telomere length, as observed in TRF assays, might be due to changes in telomere-binding proteins, which are essential to preserve functional telomeres. The TTAGGG repeats of human telomeric DNA recruit telomere specific proteins, among them the classical telomere repeat binding proteins TRF1 and TRF2 [26–31]. These proteins are known to act in *cis* to repress telomere elongation. Whereas telomerase itself seems to be the target of TRF1-driven regulation of telomere length, TRF2 targeting and telomerase inhibition have been shown to induce additive effects on telomere length, thus suggesting that TRF2 can exclusively activate a telomeric degradation pathway. Accordingly, overexpression of full-length TRF2 leads to an increased rate of telomere shortening. TRF1 and TRF2, together with TRF1- and TRF2-interacting ankyrin-related ADP-ribose polymerase (Tankyrase) as well as TRF1-interacting nuclear protein 2 (TIN2), reorganize the linear chromosome end into a higher order structure known as the T-loop, a protected structure that hides the very end of the chromosome [30, 31, 39–45]. The telomere regulator, RAP1, was identified as a protein that negatively regulates telomere length by specifically interacting with TRF2 while functioning as a protein adaptor that brings different factors into the telomeric complex [32–35]. Thus, whereas the C-terminal domain of RAP1 (RCT) functions to interact with TRF2 and tethers RAP1 to telomeres, the N-terminal BRCT, Myb and linked domains of RAP1 have been suggested to recruit one or more protein factors, which together are required for the actual execution of the negative control of telomere length [33, 34].

What are the negative regulators recruited by the TRF1-RAP1 complex at chromosome ends? TRF2 and RAP1 have been shown to associate with proteins that play important roles in DSB repair, including components of the MRE11 complex (MRE11, RAD50, and NSB1) [34, 46]. TRF2/RAP1-dependent telomere maintenance is influenced also by components of the DNA damage response pathway including Ku70/Ku80 and PARP1 [47–50]. Our current findings strongly suggest that HRGβ2 might play a previously-unrecognized role in stabilizing the TRF2/RAP1 complex at chromosome ends which, in turn, might account for HRGβ2's ability to regulate telomere length maintenance in human cancer cells. While no significant changes were found in the expression of telomere-associated proteins, such as TRF1, TIN2, Tankyrase and Ku70/Ku80, in response to changes in HRGβ2 expression (Figure 3, and data not shown), HRGβ2 overexpression induced a marked up-regulation of TRF2 and RAP1 and, in parallel, promoted telomere shortening. Moreover, knockdown of endogenous HRGβ2, either by stably expressing an antisense HRGβ2 cDNA or transfection with an HRGβ2-targeted siRNA, significantly diminished the abundance of TRF2 and RAP1, reduced the telomeric signals of these factors, and concomitantly promoted telomere lengthening. Although the precise mechanism responsible for the HRGβ2-driven regulation of native TRF2 and RAP1 remains to be elucidated, it is reasonable to postulate that altered post-translational modifications and, most likely, protein-protein interactions, might underlie the ability of HRGβ2 to regulate the TRF2/RAP1 complex. Consistent with this notion, HRGβ2 significantly co-localized with TRF2 and RAP1 at the telomere complex (Figure 5), while co-immunoprecipitation assays revealed that HRGβ2 could directly interact with TRF2 (unpublished observations).

Short telomeres have a dual role in carcinogenesis since they could potentially lead to both cancer protection and cancer promotion [51, 52]. Whereas telomere attrition due to the absence of telomerase activity (for instance, *Terc* or *Tert* knock-out mice) has been shown to suppress tumor growth [52–54], short telomeres have been described to generate chromosome instability, which in turn may lead to an increased risk of developing several cancers [55, 56]. Significant telomere shortening is prevalent in pre-invasive breast lesions (e.g., ductal carcinoma *in situ*) as well as in focal areas of histologically-normal epithelium, where breast carcinoma is thought to arise [57, 58]. Moreover, reduced telomere DNA content correlates with genomic instability and metastasis in invasive breast carcinomas [59, 60]. Consequently, telomere shortening has become a strong candidate as a cause of structural chromosome defects contributing to BC development and progression. Our findings are compatible with an HRG-regulated and TRF2/RAP1-dependent maintenance of telomere homeostasis, which depends on the action of telomerase at telomeres [61]. Interference with endogenous HRGβ2 might prevent the TRF2/RAP1 complex from sequestering telomere ends, thus enabling access to telomerase and resulting in extended telomeres. Conversely, HRGβ2 overexpression would promote the up-regulation and stabilization of the TRF2/RAP1 complex at chromosome ends, thus impeding telomerase access and resulting in shortened telomeres. It could be argued that short telomeres should trigger a DNA-damaging response (DDR), which, if not abrogated, could act as a tumor suppressor mechanism. This short telomere-triggered anti-cancer mechanism, however, can only occur in telomerase-deficient cells as previously demonstrated in the telomerase-deficient mouse model, which is resistant to carcinogenic treatments [53]. The cancer cell models used in this study are wild-type for telomerase genes and therefore are able to elongate short telomeres, thus ensuring continuous cell division.

According to the “protein-counting” model [28–31, 61], HRGβ2-promoted very short telomeres might not bind sufficient amounts of TRF2 to create a “closed state” at the telomere [28–31, 61, 62]. At this stage, telomere elongation would again take place, preventing a complete loss of the telomeric DNA and resulting in the stabilization of the telomeres at a shorter length in HRGβ2-overexpressing cancer cells. The cancer promoting actions of HRGβ2 could therefore depend on its ability to trigger and maintain telomere shortening in a TRF2-dependent manner. Consistent with this concept, short telomeres produced by TRF2 overexpression have been shown to correlate with promotion of tumorigenesis [55, 56]. Furthermore, an increased chromosomal instability due to elevated TRF2 can promote tumorigenesis in the presence of normal levels of telomerase [55, 56]. Not surprisingly, TRF2 overexpression can be found in a high percentage of human basal cell carcinomas as well as in other types of human cancer including breast tumors, lung carcinomas, gastric carcinomas and liver hepatocarcinomas [63–65]. TRF2 overexpression in human hepatocarcinomas correlates with progressive telomere shortening, further suggesting a key role for TRF2 in linking telomere length and cancer progression [65]. Increased TRF2 expression leads to increased chromosomal instability due to telomere uncapping resulting from a critical telomere shortening and a defective nucleotide excision repair (NER) pathway [56]. We acknowledge that our study did not evaluate whether, in the setting of abrogate DNA damage checkpoints, HRGβ2-induced shorter telomeres concomitantly increased chromosomal aberrations due to end-to-end chromosomal fusions and subsequent breakage and rearrangement in cancer cells. Although further studies should elucidate how HRGβ2 impacts telomere-related chromosomal instability and DDR signaling, it is worthwhile to note that HRGβ2 overexpression dramatically increases the sensitivity of human cancer cells to DNA damaging agents such as doxorubicin and etopoxide [6, 12, 13], thus recapitulating the increased sensitivity to DSB inducers and ionizing radiations promoted by short telomeres [20, 21, 36, 37, 66].

In summary, our data establish for the first time a functional link between HRGβ2 and telomere length maintenance through the unexpected ability of HRGβ2 to specifically regulate and interact with telomeres in human cancer cells. Since little is known about the factors involved in the regulation or in the recruitment of telomere-binding proteins to telomeres during normal development and in pathological conditions, our current findings demonstrating how the nuclear function of HRG unexpectedly involves the alteration of telomere homeostasis *via* TRF2 and RAP1, adds a further tier of complexity to the regulatory mechanisms of telomere length and function during tumor initiation and metastasis.

## MATERIALS AND METHODS

### Materials

Primary antibodies against TRF2 (IMG-124), TRF1 (IMG-283) and RAP1 (IMG-272) were purchased from Imgenex (San Diego, CA). The primary rabbit polyclonal anti-HRGβ_2_ antibody (C-20; sc-348) was purchased from Santa Cruz Biotech. (Santa Cruz, CA). Recombinant HRG was purchased from Lab Vision Corporation (Fremont, CA).

### Cell lines and culture conditions

MCF-7, MDA-MB-231 and Hs578T human BC cells and A-431 human epidermoid carcinoma cells were obtained from the American Type Culture Collection (ATCC). Cells were routinely maintained in phenol red-containing improved MEM (IMEM, Biosource International, Camarillo, CA) supplemented with 5% (v/v) fetal bovine serum and 2 mmol/L L-glutamine at 37°C in a humidified atmosphere of 95% air and 5% CO_2_, unless otherwise specified. HRGβ_2_-overexpressing T6 and T8 clones were engineered by transfecting MCF-7 cells either with an empty eukaryotic expression vector pRC/CMV (MCF-7/V cells) or with pRC/CMV containing the full-length cDNA of human HRGβ_2_ as described [9]. HRGβ_2_-overexpressing MCF-7/T6 and MCF-7/T8 clones were grown as indicated above, except that 450 μg/ml G418 (Geneticin, Sigma-Chemicals, St. Louis, MO) was added to the culture medium. MDA-MB-231 and Hs578T antisense (AS)-HRGβ_2_ clones were generated as described [11]. Briefly, the HRGβ_2_ cDNA (amino acids 1-426) was cloned in an antisense direction into pRC/CMV and subsequently transfected into MDA-MB-231 and Hs578T cells. MDA-MB-231/AS-V and Hs578T/AS-V cells were transfected with empty pRC/CMV, whereas MDA-MB-231 AS-HRG and Hs578T AS-HRGβ_2_ cells were transfected with pRC/CMV containing the antisense-HRGβ_2_. Selected AS-HRGβ_2_ clones and non-selected AS-HRGβ_2_ pools were maintained as described above with the addition of 200 μg/mL G418 in the medium.

### DNA constructs and retroviral infection

The PCR product generated using the HRGβ_2_ cDNA accession number 183996 as a template was cloned into the retroviral expression vector pBABE-Puro using *Bam*HI and *Eco*RI restriction sites. Full-length HRGβ_2_ cDNA in pBABE was transfected into a high efficiency transient packaging system using FuGENE transfection reagent (Roche, Indianapolis, IN). Retrovirus-containing medium collected after 48 h was used to infect MCF-7 cells for 24 h in the presence of Polybrene (Sigma-Chemicals). Infected MCF-7 cells were grown for an additional 24 h in standard medium and stable cell lines (MCF-7/pBABE and MCF-7/HRG) were selected and expanded in the presence of 2.5 μg/mL puromycin for at least two weeks. HRGβ_2_ expression was assessed by RT-PCR using the Gene Amp Kit (Promega Corporation, Madison WI).

### Analysis of telomerase activity (TRAP assay)

Telomerase activity was determined by using the highly sensitive one buffer, two enzymes *in vitro* system TRAP_EZE_^®^ Telomerase Detection Kit (Intergen, Purchase, NY) [36, 37]. In the first step of the reaction, telomerase adds a number of telomeric repeats (GGTTAG) onto the 3′ end of a substrate oligonucleotide (TS). In the second step, the extended products are amplified by PCR using the TS and RP (reverse) primers, thus generating a ladder of products with 6 base increments starting at 50 nucleotides. Additionally, each reaction mixture contains a primer (K1) and a template (TSK1) for amplification of a 36-bp internal standard. In our experiments, sub-confluent cells were collected by trypsinization and counted using the Coulter counter. Lysates from 10^3^ cells were incubated in a telomerase reaction buffer at 30°C for 30 min, followed by 33 PCR cycles (94°C/30 seconds, 59°C/30 seconds). The PCR products were resolved on a 12% polyacrylamide gel, visualized with SYBR Green I Nucleic Acid Gel Stain, and scanned directly with a Molecular Dynamics Storm System (Molecular Dynamics). Relative telomerase activities were obtained after comparison with the signal from parental cells, which were always measured on the same gel. Between two and five parallel measurements were performed from each clone.

### Analysis of terminal restriction fragment (TRF) length

Cells from a sub-confluent 100-mm diameter culture dish were harvested by trypsinization, washed in cold PBS, collected by centrifugation for 5 min at 1,500 *x* rpm, and counted using the Coulter counter. Cell pellets were frozen at −80°C until analysis. Genomic DNA was isolated from cell pellets (approximately 5 × 10^6^ cells for each clone) using a DNA extraction kit (Qiagen, Santa Clarita, CA) with inclusion of an RNase A digestion step, and quantitated with a spectrophotometer. Protein-free DNA was cleaved with *Hinf*I and *Rsa*I restriction enzymes (Gibco/BRL), extracted once with phenol/chloroform/isoamyl alcohol, ethanol precipitated, and resuspended in Tris-EDTA to generate the TRF. A portion of DNA was subjected to electrophoresis in a 0.5% agarose gel in 1X Tris-borate EDTA (TBE) buffer at 2 V cm^−1^ for 17 h. The gel was subsequently dried at 60°C for 2 h, denatured for 30-60 min in 0.5 mol/L NaOH and 1.5 mol/L NaCl, and neutralized for 30-60 min in 1 mol/L Tris-HCl, pH 8.0 and 1.5 mol/L NaCl. The dried gel was then subjected to in-gel hybridization with a [γ-^32^P]-ATP 5′ end-labeled telomeric oligonucleotide probe [γ-^32^P-(TTAGG)_3_] [36, 37].

### Calculation of mean TRF length

Although an estimate of the TRF length can be obtained by visually comparing the size of the signal smear with molecular weight markers, we calculated the TRF length for each sample by integrating the signal intensity above background over the entire TRF distribution as a function of TRF length using the formula:

L=∑(ODi •Li)/ ∑(ODi) Equation 1

Where ODi and Li were the signal intensity and TRF length, respectively, at position i on the gel image. This method takes into account the greater intensity of signals from larger fragments. The amount of telomeric DNA was calculated by integrating the volume of each smear using a Phosphorimager (Amersham Biosciences) and Image-QuaNT software (Molecular Dynamics). Briefly, the scanned image was divided into a grid consisting of *X* columns and multiple rows where *X* denotes the number of samples. The rows were positioned to cover the entire vertical length of the image. The vertical size of grid boxes was arbitrary, but it was small enough such that many boxes overlaid a signal smear. The background was calculated using two rows above and two rows below each signal smear. The background OD was averaged and then subtracted from the OD of each grid box to give the signal due to TRFs at that position. For each sample, OD and L were computed for each grid box, where OD was the total signal intensity within a grid box and L was the distance (in cm) at the mid-point of the grid box. Mean TRF length was then calculated using *Equation 1* and values were reported as the average of at least two independent Southern blots [36, 37].

### Calculation of population doublings (PD)

The total number of cells harvested was calculated at every subculture and the number of accumulated PDs per passage was determined using the equation PD = (*A/B*)/log 2, where *A* is the number of harvested cells and *B* the number of plated cells, not corrected for plating efficiency [36, 37].

### Western blot analyses

Harvested cells were washed twice with PBS and then lysed in buffer [20 mmol/L Tris (pH 7.5), 150 mmol/L NaCl, 1 mmol/L EDTA, 1 mmol/L EGTA, 1% Triton X-100, 2.5 mmol/L sodium pyrophosphate, 1 mmol/L β-glycerolphosphate, 1 mmol/L Na_3_VO_4_, 1 μg/mL leupeptin, 1 mmol/L phenylmethylsulfonylfluoride] for 30 min on ice. The lysates were cleared by centrifugation in an eppendorf microcentrifuge (15 min at 14,000 *x* rpm, 4°C). Protein content was determined against a standardized control using the Pierce Protein Assay Kit (Rockford, IL). Equal amounts of protein were resuspended in 5X Laemmli sample buffer for 10 min at 70°C, subjected to electrophoresis on 10% SDS-PAGE gels and transferred to nitrocellulose membranes. Nonspecific binding was minimized by blocking membranes for 1 hr at room temperature (RT) with TBS-T [25 mmol/L Tris-HCl, 150 mmol/L NaCl (pH 7.5), and 0.05% Tween 20] containing 5% (w/v) nonfat dry milk. Membranes were then washed in TBS-T and incubated with primary antibodies for 2 hr at RT in TBS-T containing 1% (w/v) nonfat dry milk. After a further in TBS-T, anti-rabbit or anti-mouse horseradish peroxidase-conjugated secondary antibodies (Jackson ImmunoResearch Labs, West Grove, PA) in TBS-T were added for 1 hr and immunoreactive bands were detected by enhanced chemiluminescence reagent (Pierce, Rockford, IL). Blots were re-probed with an antibody for β-actin to control for protein loading and transfer. The results were scanned and intensity bands were quantified using NIH Image 1.62 software.

### Indirect immunofluorescence (IF)

Cells grown on coverslips were fixed/permeabilized with 0.2% Triton X-100/2% paraformaldehyde and permeabilized again in 100% methanol for 10 min at −20°C. Coverslips were subsequently blocked for 30 min at 37°C in 5% BSA in PBS and incubated with primary and fluorescent-conjugated secondary antibodies for 1 h each at RT. DNA was stained with diamidino-2-phenylindole (DAPI). Stained cells were observed and analyzed using a Nikon Eclipse E800 microscope equipped for epifluorescence.

### IF staining combined with telomere fluorescence *in situ* hybridization (FISH)

IF combined with FISH was performed essentially as described by Ohki & Ishikawa [38] with some modifications. Briefly, cells grown on cover-slides were fixed for 15 min in PBS containing 2% paraformaldehyde and then permeabilized in PBS containing 0.2% Triton X-100 for 5 min, washed with PBS, and post-fixed for 20 min in 100% methanol at −20°C. After two washes with PBS, cells were blocked in PBS containing 5 mg/mL BSA and 20 mmol/L glycine for 30 min. Cells were stained with anti-HRGβ_2_ antibody (1:100 dilution; C-20, Santa Cruz Biotech.) followed by TRITC-conjugated anti-rabbit IgG antibody, both of which were diluted with PBS containing 5 mg/mL BSA. Then, the cells were fixed with 4% paraformaldehyde for 7 min and incubated in PBS containing 5 mg/ml BSA and 20 mmol/L glycine for 30 min, and subjected to telomere FISH using the Telomere PNA Kit (DakoCytomation, Denmark) following the manufacturer's instructions except that cells were not pre-treated with proteolytic enzyme solution. DNA was stained with DAPI. Stained cells were observed and analyzed as above.
